# Do Physicians Have a Duty to Discuss Expanded Access to Investigational Drugs with their Patients? A Normative Analysis

**DOI:** 10.1017/jme.2023.53

**Published:** 2023

**Authors:** Stefan F. Vermeulen, Marjolijn Hordijk, Ruben J. Visser, Eline M. Bunnik

**Affiliations:** 1: DEPARTMENT OF MEDICAL ETHICS, PHILOSOPHY AND HISTORY OF MEDICINE, ERASMUS MC, UNIVERSITY MEDICAL CENTRE ROTTERDAM, THE NETHERLANDS; 2: GGZ BREBURG, TILBURG, THE NETHERLANDS

**Keywords:** Expanded Access, Treatment Options, Investigational Treatments, Professional Obligations, Moral Obligations, Shared Decision Making

## Abstract

Drawing on ethical and legal frameworks in the Netherlands, the United States and France, we examine whether physicians are expected to inform patients about potentially relevant opportunities for expanded access to investigational drugs. While we found no definitive legal obligation, we argue that physicians have a moral obligation to discuss opportunities for expanded access with patients who have run out of treatment options to prevent inequality, to promote autonomy, and to achieve beneficence.

Every day, physicians are informing patients that the end of a standard treatment trajectory has been reached, or that there are no effective standard treatment options available for their disease at all. This is tragic for all patients, but particularly so for patients suffering from lethal or severely debilitating diseases, such as end-stage metastatic disease, or inherited congenital disorders, such as cystic fibrosis,^1^ primary immunodeficiencies,^2^ or neuromuscular disorders.^3^ These tragedies take place on a daily basis in hospitals around the world; for example, over the course of 2019, 46,990 people in the Netherlands,^4^ and 599,601 in the United States (US) died of cancer.^5^ At some point during their illness, these patients will have had conversations with their doctors in which they will have been told that standard treatment options had been exhausted.

Sometimes, for these patients, hope of a cure, alleviation of symptoms or prolongation of life may come in the form of an unapproved, investigational treatment. Patients may be invited to try an investigational drug in the context of a clinical trial. However, when patients cannot enroll in trials, due to their geographic location or because they do not meet the enrollment criteria of ongoing trials, they may qualify for expanded access to investigational drugs, which is also referred to in the literature as early access, managed access, pre-approval access, or compassionate use.^6^

Expanded access regulations allow physicians to prescribe investigational drugs outside of clinical trials, under strict conditions.^7^ Although regulations differ across countries,^8^ to be eligible for expanded access, patients must generally fulfil three criteria: a) the disease must be serious or life-threatening, b) there must be no suitable approved treatments left and c) there must be no clinical trials in which patients can logistically or reasonably participate.^9^

The uptake of expanded access programs differs between countries, with the number of annual applications ranging from 100-200 patients per year in the Netherlands,^10^ and 1000 in the US,^11^ to over 20,000 in France,^12^ and Turkey,^13^ although it should be noted that the numbers may recently have increased due to Covid-19-related expanded access programs.^14^ The very few empirical studies conducted so far suggest that in some countries, practical and moral concerns withhold physicians from pursuing expanded access to investigational drugs in practice.^15^

Expanded access to investigational drugs is not a standard practice in clinical care. Therefore, treating physicians may not always suggest or pursue opportunities for expanded access on their own initiative. On the one hand, physicians have reason *not* to do so, for the drugs’ safety and effectiveness have not been definitively proven, the drugs have not been approved for marketing, and their use is not recommended by clinical guidelines or as part of standard of care. On the other hand, it could be argued that when patients have run out of standard treatment options, cannot be enrolled in clinical trials, and expanded access to an investigational drug could be the only potentially curative or life-prolonging treatment option left for them, they have a right to be told about that *ultimum remedium*. Should doctors inform their patients about existing opportunities for expanded access to investigational treatments?

This paper examines whether physicians have a duty to discuss expanded access with their patients. To our knowledge, there are no international guidelines on physicians’ informational obligations concerning expanded access to investigational treatments. In addition, professional medical-ethical codes fail to address the issue of expanded access to investigational drugs. These obligations are likely determined by national ethico-legal frameworks that vary from country to country. We attempt to investigate if there could be a legislative or moral norm underlying information provision regarding expanded access, especially in the light of highly varying uptake levels in different countries.

This paper examines whether physicians have a duty to discuss expanded access with their patients. To our knowledge, there are no international guidelines on physicians’ informational obligations concerning expanded access to investigational treatments. In addition, professional medical-ethical codes fail to address the issue of expanded access to investigational drugs.^16^ These obligations are likely determined by national ethico-legal frameworks that vary from country to country. We attempt to investigate if there could be a legislative or moral norm underlying information provision regarding expanded access, especially in the light of highly varying uptake levels in different countries. Drawing on ethico-legal provisions in our home country, the Netherlands, we assess the scope of physicians’ duties in relation to information provision about non-standard treatment options in the Netherlands, and briefly compare the Dutch framework to legal frameworks in two other countries: France, which has a relatively high uptake of expanded access,^17^ and the US, which has seen recent regulatory reform in the 21st Century Cures act and ‘Right-to-Try’ legislation that might influence practices of information provision.^18^ Then, we investigate, from an ethical point of view, whether treating physicians have a *moral* duty to discuss existing opportunities for investigational treatments with their patients.

## Ethico-Legal Framework in the Netherlands

The Dutch Medical Treatment Act (*Wet op geneeskundige behandelingsovereenkomst*, WGBO) sets forth legal provisions in relation to information provision and informed consent within the physician-patient relationship. It points out the elements to be discussed in conversations between doctors and patients about medical treatments. While it does not contain any specific provisions regarding expanded access, it does require physicians to inform patients about “other methods of examination or treatment *that are applicable*” [emphasis added].^19^ The Royal Dutch Medical Association (*Koninklijke Nederlandsche Maatschappij tot bevordering der Geneeskunst,* KNMG), in its interpretation of the Act, indicates that a treatment option is an applicable alternative if the following criteria are met: the option a) is accessible to the patient, b) falls within the competency domain of the physician, c) is relevant to treating the disease of the patient, and d) is not associated with “ethically unacceptable” implications.^20^ The KNMG interpretation is considered to be the professional standard on information provision. If existing opportunities for expanded access to investigational treatments satisfy these four criteria, it follows that physicians may have a duty to inform patients about such opportunities. Below, we discuss the applicability of these criteria one by one.

Firstly, for a medical treatment to be accessible, it is required that the treatment can be brought to patients who are in medical need of it. Although regulations allow for expanded access to investigational drugs, and expanded access programs are set up for some investigational treatments to provide access to groups of patients, it does not always seem possible to obtain access in practice. An interview study among medical specialists in The Netherlands, Turkey and the United States (US) reports that accessibility to these programs is hampered by practical constraints, including lack of funding, demands on time and effort, and limited supply.^21^ Furthermore, in most countries, including the Netherlands, many other European countries, and the US, there is no routine reimbursement of expanded access. This means that pharmaceutical companies, hospitals, or health insurers would have to pay for the cost of treatment, and neither of them may be willing or able to do so.^22^ This implies that doctors who initiate a process of requesting expanded access, may not always be successful in obtaining the investigational treatment for their patients, and consequently, that the investigational treatment is not always accessible to patients.

Secondly, it is not self-evident that physicians are competent to prescribe investigational treatments via expanded access programs, even when these treatments fall within their medical specialty. Medical specialists are expected to pursue lifelong learning and keep their knowledge and skills up to date, through scientific literature, professional organizations, and conferences, as well as training. They should therefore theoretically know about and be able to prescribe investigational treatments that fall within their medical specialty. Yet, they may feel ill-equipped in practice. As the process of clinical research on the unapproved treatment has not yet been concluded, and as appropriate application of the drug will not have been detailed in clinical guidelines, the physician will have less guidance and experience at his or her disposal on the use of the drug and on the management of the patient, than is the case with approved treatments.

Thirdly, it may be difficult for treating physicians to establish the relevance of investigational drugs for individual patients. After marketing approval, the label on a drug and clinical guidelines set forth the indication(s) for which the drug may be used. In the pre-approval setting, however, it may not be clear how future indications for the drug will be defined, and doctors may not be certain whether an investigational drug is relevant for an individual patient. While it is likely that the future label of a new drug will reflect the enrollment criteria of the clinical trials conducted thus far, in practice, doubts about the efficacy and safety of the investigational drugs and concerns regarding its practical feasibility may lead doctors to be uncertain about the relevance of an expanded access option.^23^

Fourthly and finally, and in contrast to the first three criteria, the fourth criterion is formulated negatively, demanding the absence of unacceptable ethical objections. Some physicians have principled ethical objections to expanded access to investigational drugs,^24^ which may be grounded on the principle of non-maleficence:^25^ to protect patients against the risks and side effects of unproven medical treatments. Dutch physicians have also been reported to worry about the opportunity costs of expanded access: with the uncertainty surrounding its balance of risks and benefits, expanded access might reduce the patient’s quality of life and may stand in the way of a good end of life.^26^

Overall, it is unclear whether an opportunity for expanded access to an unapproved, investigational treatment will satisfy the criteria of the KNMG. While it should be noted that doctors are expected to answer questions raised by patients about standard and non-standard treatment options, the Dutch legal framework does not establish a general informational obligation regarding expanded access.

## Ethico-Legal Frameworks in Other Countries

France has no specific legislation that mentions any obligation to inform patients about expanded access. The Medical Code of the French Medical Council however does contain guidelines, which are negatively formulated and specify what types of treatment options should *not* be discussed with patients. Article 39 of the Medical Code, for instance, prohibits the presentation by physicians of “insufficiently tested procedures” as beneficial,^27^ and article 14 states that a “doctor must not divulge to the medical community a new diagnostic procedure or inadequately proven course of treatment without making the necessary reservations. He must make no such disclosures outside the medical community.”^28^ French doctors, the code holds, should therefore *not* discuss, with patients, procedures that are not sufficiently proven. When taken literally, the code of the French Medical Council might imply that physicians should not discuss expanded access with their patients as the efficacy and safety of investigational drugs can be considered “insufficiently proven”. This is striking, as, at the same time, France is known for its relatively high uptake of expanded access, and the French government provides routine reimbursement of expanded access through its *Autorisations Temporaires d’Utilisation* (ATU) program.^29^ This may suggest that in France, expanded access is perhaps generally considered more of a standard intervention in clinical practice than it is in the Netherlands.

In the US, since 2016, the passing of the 21st Century Cures Act,^30^ and, in 2018, the “Right-to-try” act,^31^ have led to increased public awareness of expanded access as compared to other countries. However, reports describe various forms of criticism of both regulatory and practical inefficiencies that render expanded access less feasible for physicians.^32^ The American Medical Association’s (AMA) Code of Medical Ethics currently makes explicit reference to information provision about expanded access.^33^ In *Opinion E-7.3.10*, it describes a positive obligation for physicians to look into expanded access and determine whether it is applicable for their patients: “Physicians who care for patients with serious, life-threatening illness for whom standard therapies have failed, are unlikely to be effective, or do not exist should determine whether questions about access to investigational therapy through the U.S. Food and Drug Administration’s “expanded access” program are likely to arise in their clinical practice. If so, physicians should familiarize themselves with the program to be better able to engage in shared decision making with patients.”^34^ This implies that physicians should be prepared to respond to questions raised by patients about expanded access but does not require them to bring up existing opportunities on their own initiative.

## Is There a Moral Duty to Inform? Arguments in Favor

While our brief analysis of ethico-legal frameworks suggests that in the Netherlands, France and the US, there is no clear legal obligation to actively inform patients about expanded access options, treating physicians may have a moral duty to do so. We will first discuss three main ethical arguments in favor of a duty to inform: to offer a chance at medical benefit, to promote autonomy, and to promote equality between patients. We will then discuss arguments against a duty to inform.

### Offering a Chance at Benefit

The founding fathers of principlism in medical ethics, Beauchamp and Childress, discern four core medical principles that should guide physicians when making treatment decisions for, and with, their patients: the principles of respect for autonomy, non-maleficence, beneficence, and justice.^35^ Expanded access to investigational drugs is primarily intended to provide patients a final chance at medical benefit, in line with the principle of beneficence. It is intended as therapeutic.

However, both the chances at benefit and the intended benefit can be small, or very small. Due to the nature of expanded access to investigational drugs, as said, the safety and efficacy of treatments have not been proven definitively. Expanded access is often requested to investigational drugs that are in late phase II, phase III or post-phase III of the clinical development trajectory.^36^ Drugs that are further in the development process have an increasingly higher chance of reaching marketing authorization, with over 60% of drugs in phase III eventually approved for marketing.^37^ But even if they do, the added benefit of many newly registered drugs is disputed in literature.^38^

At the same time, the expanded access programs are only open for patients in ‘back against the wall’ situations. To provide these patients with a last chance to achieve curative or life-prolonging treatment they have to be informed about the opportunities, for which they have no other way.

### Promoting Autonomy

Another reason in favor of a duty to inform patients about expanded access is that it helps to respect patient autonomy, one of the other principles put forward by Beauchamp and Childress.^39^ The principle of respect for autonomy is operationalized in clinical practice, among other things, in the ethico-legal requirement of informed consent. Based on adequate information, competent patients should be able to decide freely about medical treatments and provide their consent. Only if patients know about the possibility of expanded access, will they be able to decide whether they wish to pursue it.

Over the years, various standards have been used for the disclosure of information by physicians as part of the informed consent process: a professional standard, a reasonable person standard, and a subjective standard. Traditionally, the professional standard of disclosure applied, which stated that doctors should inform patients about that which (other) physicians would consider relevant based on their professional experience and expertise. This standard was re-evaluated as too limited and paternalistic, and has been replaced over time by the ‘reasonable person’ standard for disclosure, which is based on what a reasonable person would need to know in order to make an informed decision.^40^ This was part of a shift in many countries towards a form of disclosure in which the physician was seen more as an expert-advisor instead of a sole decision-maker. These standards are clearly represented in court cases of negligence and medical malpractice suits in the United States. Beauchamp and Faden describe how in the past, these cases were often decided by having physicians testify, representing the professional standard of disclosure.^41^ Later, however, the jury, made up of citizens, functioned as “reasonable person standard” to decide on the adequacy of disclosure. Today, the current standard for information provision is the subjective standard of disclosure, in which the extent of information provided should be determined by what the individual patient needs to know. To ensure informed consent, the subjective standard demands that treating physicians engage in dialogue with their patients, to determine and meet individual patients’ informational needs and preferences.

In order to successfully engage in shared decision-making, there should be no information-asymmetry between both participants. This means that to respect the autonomy of eligible patients, physicians should indeed disclose information about potentially relevant opportunities for expanded access.

Note that these standards of disclosure are commonly applied to the extent, the amount and the level of detail, of the information provided about treatment options — the “scale” of disclosure. Our question concerns rather the “scope” of disclosure: Should a particular opportunity to pursue expanded access to a particular medical treatment be brought up as an option? Using a professional standard, expanded access may fall outside of the scope of informational obligations: physicians may consider it insufficiently relevant or accessible, or beyond their competency, or may have general moral concerns about expanded access. Using a reasonable person standard to determine the scope of information to be provided, however, would mean that a physician should disclose all the treatment options (including non-standard treatment options) that a reasonable person would want to be informed about to make a decision about medical treatment. As expanded access is often considered a patient’s final curative or life-prolonging option, a reasonable person — and most patients — would desire to know about a relevant opportunity to pursue expanded access to an investigational treatment.^42^ Using a subjective standard would further imply that the individual patient should determine how much and what kinds of information he or she wishes to receive about this option prior to deciding about treatment.

The individual patient may have good reasons to renounce such opportunities (e.g. given the safety risks involved), and, needless to say, their autonomous decision not to pursue expanded access should be respected. But withholding the information *that this option exists*, is not in line with the principle of respect for autonomy. The patient cannot exercise self-determination without this information. In order to successfully engage in shared decision-making, there should be no information-asymmetry between both participants.^43^ This means that to respect the autonomy of eligible patients, physicians should indeed disclose information about potentially relevant opportunities for expanded access.

### Promoting Equality between Patients

When there is no expectation or requirement that relevant opportunities for expanded access to investigational drugs are routinely discussed with patients, the result will be that some patients are informed about expanded access, while others are not. This may run counter to the principle of justice (another principle put forward by Beauchamp and Childress), especially when the dividing lines between those who will and those who will not be informed, follow the lines of — and may reinforce — existing health disparities that are associated with structural social inequalities. While health disparities within universal access healthcare systems in high-income countries may pose less pressing problems than do current health inequalities between countries around the world and shortages of health care in low-income countries, they are nevertheless problematic.^44^

Physicians may choose not to discuss expanded access with their patients, based on medical, but also on personal moral considerations, as discussed previously. However, they will answer questions coming from patients, as is required, for instance, by the Dutch standard, and/or engage in discussions and shared decision-making about expanded access, when these are brought up by patients, as is required by the US Code. Patients differ in terms of health literacy, educational background, socio-economic status, connections, personality, et cetera. With these differences, the likelihood of them becoming informed about expanded access by themselves will differ. Assertive, well-informed patients may look up information about investigational drugs and ask their treating physicians about expanded access, while other patients who are less informed or less assertive may not. These patients may already be in a less favorable position, in terms of socio-economic status, to achieve good health outcomes. By not being informed, they are withheld both potential direct medical benefits and the benefits associated with shared decision-making about medical treatments, which, when executed optimally, is appreciated by patients,^45^ and associated with better outcomes.^46^ Inequitable access to information about expanded access may thus exacerbate existing health disparities.

This is at odds with the principle of justice, as this implies that some patients may be informed about possibly relevant treatment options — and gain access to potential benefits associated with these options — whereas others may not, based on contingent patient and physician characteristics. A general obligation to inform eligible patients about relevant opportunities for expanded access to unapproved, investigational treatments would help to promote equal access to such treatments.

## Arguments Against a Moral Duty to Inform

As mentioned, there are many ethical issues associated with expanded access to investigational drugs, including safety risks, opportunity costs, and financial barriers. These issues are raised by the uncertainties surrounding unapproved treatments and local systems for expanded access, and coupled with the principle of non-maleficence, *not* by the provision of information. These issues must be weighed against the potential benefits of the *ultimum remedium*. The weighing of benefits and risks associated with treatment options is common practice within healthcare, and takes place through exchange of information in discussions between patients and their treating physicians. There are few arguments to be made against a duty *to inform* patients about treatment options associated with risks and benefits, even if the risks are greater and the potential benefits are smaller than for standard treatments.

While there are no principled arguments against a duty to inform, there may be practical arguments against such a duty. First, it would require physicians to have extensive knowledge about new developments in their field. This would fuel the need for continuous education of physicians on expanded access and on treatments under clinical development within their respective medical specialties. It would also imply that doctors venture away from “evidence-based medicine,” defined as the prescription of approved treatments based on clinical guidelines, and develop their competencies at considering clinical research data as they emerge, real time, through published results of clinical trials. Doctors may not feel equipped to do so. Recent reports show that physicians face problems regarding the practical feasibility of expanded access, including limited access to expanded access programs and restrictive institutional policies.^47^ Second, doctors may be concerned that they will elicit only “false hope” in patients when it remains difficult to obtain access to unapproved drugs in practice.^48^ This could potentially be harmful to patients and be at odds with the principle of non-maleficence.^49^ To avert these harms, institutional change might be required, to provide treating physicians with resources, assistance and support (e.g. dedicated personnel) to facilitate expanded access in practice and render it more feasible.^50^ Such resources required may not be available in healthcare systems already under duress. Third, there are concerns that if expanded access to investigational treatments were to become more widely accessible, patients may no longer be willing to participate in clinical trials, especially if these trials involve randomization to placebo or to a standard of care that is known to be burdensome or ineffective. This would jeopardize the clinical development of new treatments and runs counter to the interests of wider or future patient populations. Note that this argument does not hold as long as the classic criteria for expanded access are met, and patients continue to be eligible for expanded access only if they cannot be enrolled in clinical trials.

## Conclusion

Physicians are uniquely positioned to know about investigational drugs that are being developed in clinical trials around the world, and about the regulations for expanded access in their respective countries. We argue that, as part of the informational obligations of physicians towards patients, expanded access should be discussed in consultations with patients who have run out of treatment options and who may be eligible for relevant expanded access programs for unapproved, investigational treatments, outside of clinical trials. To provide a chance at benefit, promote autonomy and equal treatment of patients currently facing unmet medical needs, we conclude that treating physicians should actively bring to the table relevant opportunities for expanded access to investigational medical treatments.

Our analysis suggests two priorities for further research: the position of expanded access in ethico-legal frameworks and the barriers perceived by physicians. First, we found that for expanded access to be used in practice, it does not necessarily have to be discussed in ethical codes. The French Medical Code neither discusses expanded access directly nor promotes information provision about expanded access. This is not in line with the reported high uptake of expanded access in France and its presumed acceptability and relative feasibility, considering its policy of routine reimbursement of expanded access. This might suggest that patients can make use of expanded access in health care systems that render it *practically* feasible, not necessarily in countries that have professional guidelines, regulations, and processes in place, but lack practical arrangements to make it possible. It may be difficult for physicians to navigate the expanded access landscape, as pointed out in the literature.^51^ Extra training for physicians should focus on the practical procedures of expanded access and on communication with patients about the opportunities and caveats of expanded access. Furthermore, there should be more transparency about expanded access programs for physicians and patients. Requiring manufacturers to provide information about their expanded access policies and programs, such as is done in the US 21st Century Cures act,^52^ may help establish this. Second, physicians might perceive higher barriers to obtaining expanded access than there actually are. In the US, the FDA approves more than 99% of requests submitted using expanded access regulations.^53^ Professional education might help physicians to overcome perceived and real barriers and to close the gap between theory and practice.

In this article we have argued that physicians have a moral obligation to discuss expanded access, as part of the informed consent process in decision-making about medical treatment. Our main argument is to give patients a chance — albeit small — at curative or life-prolonging treatment. Discussing the option of expanded access furthermore promotes patients’ autonomy and is something that a reasonable patient would expect from their treating physician. There could be practical issues with informing patients about expanded access to investigational drugs. We argue the process requires physicians to be to be highly informed about new developments, and would require institutional change to make expanded access a feasible option, yet we argue patients should be allowed to make decisions about expanded access themselves and cannot do so without being adequately informed about their options. Finally, a general obligation to inform eligible patients about relevant expanded access options would help remedy current inequalities between patient groups and allow all patients who have exhausted standard treatment options similar access to relevant information.
